# Editorial: Interplay between epigenetic modifiers and transcription factors in driving cancer progression

**DOI:** 10.3389/fonc.2023.1211537

**Published:** 2023-06-19

**Authors:** Yongyong Yang, Annalisa Di Ruscio, Satoshi Inoue, Guocan Wang, Shuai Gao

**Affiliations:** ^1^ Department of Urology, Feinberg School of Medicine, Northwestern University, Chicago, IL, United States; ^2^ Harvard Medical School Initiative for RNA Medicine, Harvard Medical School, Boston, MA, United States; ^3^ Department of Medicine and Cancer Research Institute, Beth Israel Deaconess Medical Center, Harvard Medical School, Boston, MA, United States; ^4^ Department of Systems Aging Science and Medicine, Tokyo Metropolitan Institute of Gerontology, Tokyo, Japan; ^5^ Division of Gene Regulation and Signal Transduction, Research Center for Genomic Medicine, Saitama Medical University, Saitama, Japan; ^6^ Department of Genitourinary Medical Oncology, The University of Texas MD Anderson Cancer Center, Houston, TX, United States; ^7^ Department of Cell Biology and Anatomy, New York Medical College, Valhalla, NY, United States; ^8^ Department of Biochemistry and Molecular Biology, New York Medical College, Valhalla, NY, United States

**Keywords:** epigenetic modulators, transcription factors, gene transcription, reprogramming, super-enhancer, liquid-like phase separation, prostate cancer, drug-resistance

Epigenetic modulators can render “on-off” switches on the chromatin by modifying their enzymatic substrates and set up a unique nuclear environment for executing the molecular actions of chromatin. Transcription factors typically bind to the chromatin *via* recognizing the cognate DNA motifs and involve epigenetic machinery to regulate gene transcription. How epigenetic modulators cooperate with transcription complexes in order to tune the oncogenic program remains largely unknown and requires further research. Many transcription factors remain undruggable, due to the lack of a targetable domain while a significant advance has been witnessed for the development of small molecules targeting various players in epigenetic modification.

In this Research Topic, both original research and reviews contribute to our further understanding of how transcription factors and epigenetic modulators orchestrate transcriptional programs that promote cancer progression, and they provide important insights into the generation of novel therapeutic strategies for treating cancers that acquire drug resistance.

Sequencing of patients samples have revealed that mutations are commonly found in both epigenetic and transcriptional factors, highlighting the importance of understanding the molecular functions of both factors in tandem, rather than in isolation. In the multiplatform analysis performed in Gastric Cancer (GC), Dong et al. identified four major genomic subtypes of GC. Several epigenetic modulators emerged as the top-ranked driver mutant genes, including: *ARID1A*, a core member of the SWI/SNF chromatin remodeling complex; *DNMT3L*, a key player in mediating DNA methylation. It will be interesting to further explore the molecular mechanisms of how these mutations drive GC progression. In the same study, the authors also identified *SMAD4* as one of the frequently mutated genes in a subgroup of GC, and seemingly playing an important role in perturbing the tumor microenvironment (TME), which warrants future functional studies to the role of *SMAD4* mutations on TME.

Genetic alterations of *CHD1* that include missense mutation, truncations, and genetic loss are observed in various cancers, with homozygous deletion being the most frequent event in prostate cancer (PCa). As an early event, *CHD1* loss has been well known to redistribute androgen receptor (AR) chromatin binding and lead to PCa tumorigenesis (1). Loss of *CHD1* was also shown to promote tumor heterogeneity during AR-targeted therapy and confer antiandrogen resistance (2). Paradoxically, CHD1 appears to be essential for the survival of PTEN-deficient prostate tumors (3). Li et al. provided a comprehensive review of CHD1 in PCa, wherein the function of *CHD1* loss can be highly dependent on the molecular background. The authors shows that *CHD1* loss synergizes with *SPOP* mutations in modulating AR signaling and DNA damage repair pathways suggesting that CHD1 can be used as a biomarker for predicting the response to AR-targeted therapies and a therapeutic target.

Transcriptional reprogramming is a common determinant for how cancer cells develop drug resistance and metastasize to secondary sites (4). As a nuclear hormone receptor, AR is frequently mutated and amplified in the late stage of PCa that has become unresponsive to the AR signaling inhibitors (ARSi), a stage known as castration-resistant prostate cancer (CRPC). Labaf et al. used a stable cell line model that expresses doxycycline-inducible overexpression of AR to characterize the cistromes of AR in contexts that could recapitulate pre- and post-castration (i.e., CRPC) conditions. They found that AR in CRPC cells show a distinct transcription program enriched for DNA damage repair functions and this program was associated with poorer clinical outcomes in response to the first-line ARSi. Using bioinformatic tools, they found that EZH2, which has been shown to function as a coactivator of AR in CRPC independent of its canonical histone methyltransferase activity, emerged as the top hit to cooperate with overexpressed AR. Validation studies suggested that enhanced interaction between AR and EZH2 under the castrated condition could contribute to drug resistance in PCa patients and should be exploited by deploying both EZH2 inhibitors and DNA-damaging agents.

The acquired oncogenic transcription programs during drug resistance development require the pertinent chromatin stage (5, 6). Activation of Super-Enhancers (SEs) that drive robust oncogene expression has been shown to be a major mechanism contributing to drug resistance in multiple cancer models. The cooperation of master transcription factors and epigenetic modulators is a prerequisite for activating and maintaining the oncogenic SEs (7). To understand the molecular mechanism, attentions have been drawn to investigating the potential contribution of liquid-like phase separation (LLPS), in which high-concentration chromatin-associated factors are formed into biomacromolecular condensates (8). The multivalent interactions mediated by the intrinsically disordered regions (IDRs) are typically responsible for inducing LLPS formation (9). Targeting the SEs *via* disrupting LLPS could potentially be a potent therapeutic strategy for drug-resistant cancers. In this Research Topic, Takayama and Inoue provided a thorough review on LLPS mediated by key oncogenic transcription factors networks, such as YAP/TAZ/TEAD in lung cancer and melanoma, AR/FOXA1/OCT4 in PCa, and summarized the molecular basis of how they drive cancer progression. All this evidence supports the authors’ proposal that targeting LLPS coalesced by the transcription factors collaborations on the SEs could be a novel route for overcoming drug resistance. Indeed, screening assays from several recent reports have identified very interesting compounds to target the abovementioned transcription factors networks, such as integrase inhibitor Elvitegravir in lung cancer and nuclear analogue Ribavirin in PCa. Another recent screening study identified compound ET516 that specifically targets AR condensation in PCa (10). A more recent study with high translational potential suggests LSD1/FOXA1/BRD4 as another key framework for activating SEs in CRPC, and targeting LSD1 synergizes with BRD4 inhibitors in repressing CRPC growth (11).

Finally, in a methodology report, Pio Fabrizio et al. use OPERA_MET-A panel NGS analysis to deeply characterize the methylation status of a list of genes that have a highly translational impact. Using gDNA extracted from lung cancer cell lines, FFPE, and frozen tissues, they corroborate the previous findings, and more importantly, their results demonstrate the feasibility of constructing NGS-based methylation analysis when the biological materials are scarce.

In conclusion, identifying the crosstalk between epigenetic and transcriptomic factors during cancer progression remains to be a dynamic theme. These works should provide researchers with important insights enabling the identification of new therapeutic targets, and subsequently developing novel therapeutic approaches. This can be accelerated by multidisciplinary collaborations integrating NGS-based multi-omic tools, biochemical, structural, and imaging analyses.

## Author contributions

SG wrote the first draft, and all authors have contributed substantial inputs and edits to this Editorial and approved it for publication. All authors contributed to the article and approved the submitted version.
